# Geopolymer Modified with Insoluble Calcite and Various Silica Fumes Originated from Different Manufacturing Processes

**DOI:** 10.3390/ma18122795

**Published:** 2025-06-13

**Authors:** Yong Xu, Xiaonan Wang, Lilin Yang, Yang Liu, Tong Gao, Han Li, Yukai Wang, Ning Xie, Jing Meng, Jinping Ou, Wenshou Wang

**Affiliations:** 1 School of Chemistry and Chemical Engineering, University of Jinan, Jinan 250022, China; 2 College of Transportation Engineering, Dalian Maritime University, Dalian 116000, China; 3 Shandong Provincial Key Laboratory of Green and Intelligent Building Materials, University of Jinan, Jinan 250022, Chinamse_xien@ujn.edu.cn (N.X.); 4 Guangdong Provincial Key Laboratory of Intelligent and Resilient Structures for Civil Engineering, Harbin Institute of Technology, Shenzhen 518055, China

**Keywords:** silica fume, geopolymer, reaction mechanism, alkali-activated materials

## Abstract

It has been proven that silica fume (SF), which is a by-product from the manufacturing of single-crystal silicon, is beneficial for enhancing the mechanical properties, durability, and workability of geopolymers, as it can be quickly dissolved and form silicate-based cementitious phases in alkaline environments. However, the reinforcement mechanism of SF on geopolymer remains unclear due to the chemical complexity of geopolymer and the variety of SF types. Additionally, the solubility of calcite in an alkali environment is quite limited, and thus the formation of the amorphous calcium-based gels will be thwarted due to the lack of soluble calcium ions. Most importantly, with the development of the single-crystal industry, the amorphous silica content, crystallinity, and trace elements of SF itself have changed, which blocks the understanding of the activation mechanism of geopolymers combined with SF and insoluble calcite. To unveil the underlying modification mechanisms of SF on geopolymer materials along with insoluble calcite, in this study, two types of SF were used as the fly ash replacement in a fly ash/limestone system to prepare geopolymer materials. The reinforcement effect significantly depends on the SF types even with similar particle size and chemical compositions. The results indicate that the mechanical properties of geopolymer materials modified with SFs are not only governed by the ratio and contents of Si, Ca, Al, and Mg in SFs but also depend on the crystallinity and activity of the SFs. The hydration products could be varied according to the reaction environment. The research results not only contribute to the optimization design and application of geopolymer materials but also pave new pathways for the upcycling use of solid wastes such as SF, low-grade fly ash, or even other aluminosilicate solid wastes to achieve sustainable development.

## 1. Introduction

Silica fume (SF), one of the silica-based solid wastes, has been widely applied as a supplementary cementitious material in concrete technology [1,2,3]. In the most recent decade, with the thriving of low-carbon cementitious materials, the investigation of using SF in low-carbon cementitious materials, such as geopolymer concrete, is burgeoning. Previous studies have demonstrated that SF can significantly enhance the compressive strength [4], impermeability [5], and durability [6] of geopolymer concrete, making it an important component in the pursuit of advanced, sustainable construction materials. It was reported that the compressive strength of SF-modified geopolymers can be three times higher than the control group by partially replacing metakaolin [7]. Searching for recent related research, it was found that applying machine learning to predict the strength of geopolymer in coal gangue systems highlights the growing demand for data-driven methods to optimize material design. Our study supplements this trend by providing insights into the mechanism of SF modification, which can serve as a foundational dataset for future machine learning models aimed at correlating SF characteristics (such as amorphous content, Mg content) with geopolymer properties [8].

Compared with traditional cement concrete, the chemical composition of geopolymer concrete is more complicated because most of them are made of various solid wastes [9,10]. As a result, the impacts of SF on the properties, microstructure evolution, and hydration mechanisms are essentially different. For example, in slag-based geopolymers, the addition of SF can significantly accelerate the growth of early strength, but meanwhile, it will also reduce the workability of the system because the reaction between SF and calcium component in slag generates additional hydrated calcium silicate gel [11]. A few studies claim that the addition of SF can enhance the workability of a fly ash-based geopolymer due to its lubricating effect [12,13,14]. However, opposite evidence has also demonstrated that the addition of SF will reduce the fluidity of fly ash-based geopolymers [15,16,17]. Although great progress has been made in the application of SF in geopolymer concrete, the mechanism of SF modification on geopolymers made of various solid wastes remains unclear.

Most importantly, with the rapid development of the single-crystal silicon industry due to the huge expansion of new energy requirements, the raw materials and manufacturing process of single-crystal silicon production have significantly changed in the past years. Compared with traditional coal reduction, various reduction agents have been used to replace coal in the SiO_2_ reduction process. Consequently, as a by-product, the chemical composition and properties of the SF will be significantly changed according to the progress of the reduction technology [18,19]. The SF produced by different production processes may vary in size, specific surface area, active/inactive silica content, and chemical compositions. All of these are crucial factors that determine the designing strategy and the final performance of the SF-modified geopolymers. Therefore, an in-depth understanding of the SF modification mechanism is of great significance for optimizing the performance of geopolymers and achieving the resource utilization of SF.

To unveil the underpinning mechanism of the SF modification on the solid waste-based geopolymer, this study investigated the synergistic modification effects of limestone and SF on the fly ash-based geopolymer. The impact of the SF properties and the synergistic effect with the limestone has been explored. By comparing the mechanical properties and microstructure characterizations, the potential hydration mechanism has been revealed. This study not only provides a scientific basis for the effective utilization of various types of SF in construction but also paves a new pathway for optimizing the application of geopolymers made of complicated solid wastes.

## 2. Materials and Methods

### 2.1. Materials

**Figure 1:**
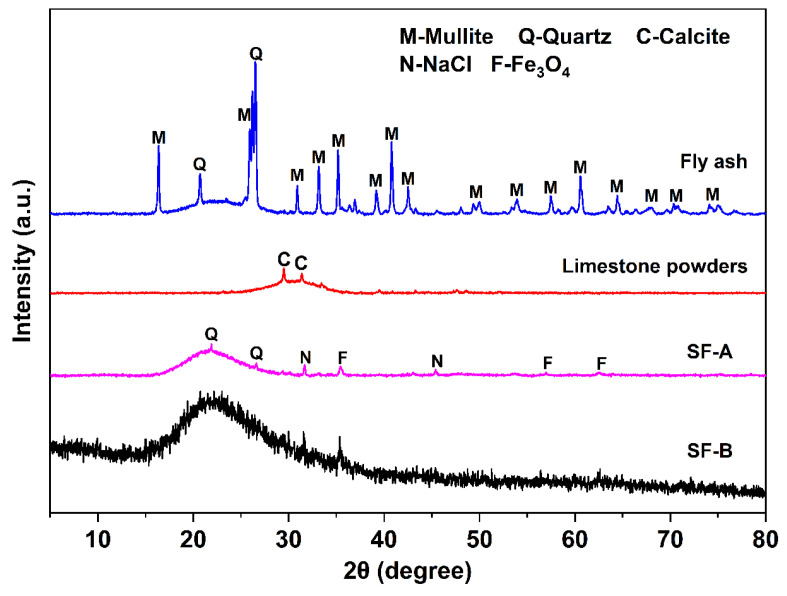
X-ray diffraction pattern of raw materials.

**Figure 2:**
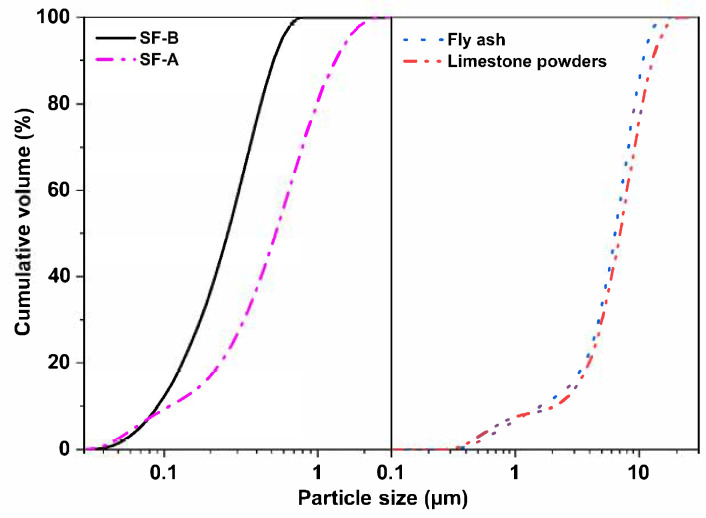
Particle size distribution of raw materials.

**Figure 3:**
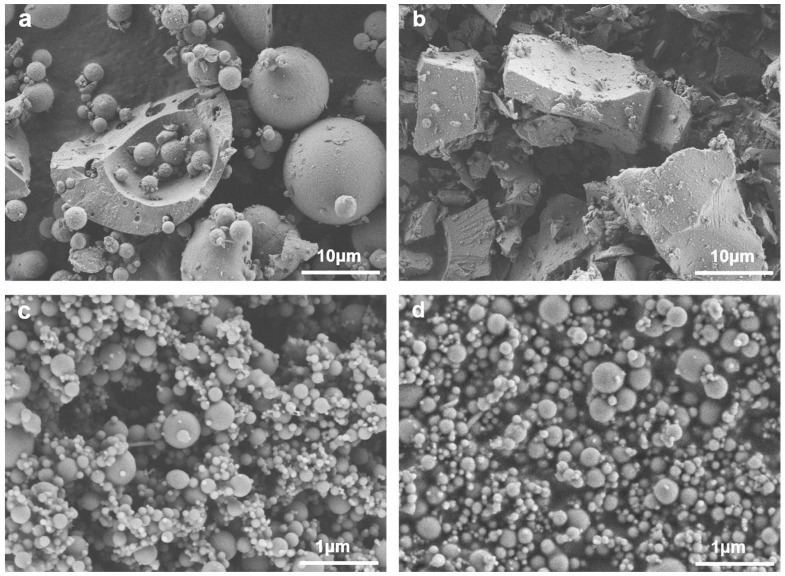
SEM of raw materials: (**a**) fly ash, (**b**) limestone powders, (**c**) SF-B, (**d**) SF-A.

The coal fly ash was obtained from the Shandong power factory. The limestone powder was provided by Longze Water Purification Materials Co., Ltd. in Gongyi City, Henan Province, China. Two types of SFs (named SF-A and SF-B), which were both by-products from the manufacturing of single-crystal silicon but with different reducing agents (coal vs. alternative agents), causing changes in crystallinity and active silica content, were obtained from Shandong Yuanheng Water Purification Materials Co., Ltd. (Rizhao, China) and Ningxia Yinuo Environmental Protection Technology Co., Ltd. (Zhongwei, China). The chemical compositions of the raw materials are listed in Table 1. As demonstrated in this table, the fly ash is mainly composed of SiO_2_ (55%), Al_2_O_3_ (30%), and CaO (4%), while the limestone is mainly composed of SiO_2_ (11%), Al_2_O_3_ (16%), and CaO (57%). The SF-A is mainly composed of SiO_2_ (80%), Fe_2_O_3_ (5%), and CaO (2%), and the SF-B is mainly composed of SiO_2_ (84%), Fe_2_O_3_ (1%), and CaO (1%). Figure 1 presents the XRD patterns of the raw materials, including the coal fly ash, limestone, SF-A, and SF-B. It can be observed that the coal fly ash is composed of quartz and mullite; while the limestone powders are mainly calcite. The SF-A is mainly composed of quartz, Hermite, and amorphous phases. In contrast, no distinctive diffraction peaks are detected in SF-B. A wide hump around 22° indicates that the main components of SF-B are amorphous phases. The average particle sizes, which were tested via a laser-particle-size analyzer (Malvern Mastersizer 3000, Malvern Panalytical, Malvern, UK), of fly ash, limestone powders, SF-A, and SF-B are 6.45, 7.03, 0.26, and 0.53 μm, respectively (as illustrated in Figure 2). The morphologies of these powders were observed through SEM images, shown in Figure 3. As demonstrated in this figure, the fly ash are mostly spherical particles with different sizes and smooth surfaces, while limestone powder particles are mostly irregular and rough. SF-A and SF-B exhibit similar spherical morphology and approximately the same particle size distribution (as shown in Figure 3c,d).

### 2.2. Mixing Proportion and Sample Preparation

To activate the fly ash, water glass (WG) with a modulus (SiO_2_/Na_2_O molar ratio) of 3.3 was used as an activator, and granular NaOH was added to adjust the modulus to 1.2. To prepare the geopolymer, the sodium silicate was first added to the mixed water and stirred, and then the granular NaOH was added. The activator remained at room temperature for 24 h. The proportion of limestone powders was kept constant at 40% of the total mass. SF-A or SF-B contents were determined by the replacement of fly ash with substitution ratios of 0%, 5%, 10%, and 15%. The mixing proportion of geopolymer is summarized in Table 2.

To reach a good dispersion effect, the SF-A and SF-B were first added to the alkali solution and ultrasonic dispersed for 5 min. During the preparation process of geopolymer, fly ash was dry stirred with limestone powders for 60 s at 2000 rpm, followed by mixing with alkali solution and stirring for another 90 s at 1500 rpm. The total water-to-precursor ratio for all samples is 0.47. The geopolymer paste was poured into a steel cube with a size of 50 × 50 × 50 mm^3^, and vibrated 60 times to remove any internal bubbles before being sealed with polyethylene film. The geopolymer paste was placed in a standard curing room at 20 °C along with a relative humidity of 99.8%. After one day, the cured sample was de-molded and cured in the standard room until it reached the testing age.

### 2.3. Test Methods

Two grams of SF-A and SF-B were completely soaked in NaOH solution with a concentration of 2.4 mol/L for 1, 4, 12, 24, 36, and 48 h. After the soaking treatment, the remained SF powders were separated by using a centrifuge. After completely drying in an oven, the mass loss proportion was calculated.

The workability was conducted according to GB/T 50448-2015 [20]. A truncated cone round die with a size of Φ 36 × 60 × 60 mm^3^ was placed horizontally in the middle of a glass plate and covered with a wet cloth. An amount of 300 g of paste was promptly filled into the truncated cone round die, and the surface was leveled by a scraper. The die was lifted vertically by 5–10 cm within 2 s and the sample was allowed to fall freely for 10–15 s. The maximum diameters of the paste were measured and the average value was calculated. The experiment was conducted twice and the average value of the two measurements was obtained with an accuracy of 0.1 mm. The compressive strength of geopolymer samples cured after 3, 7, and 28 d was tested according to the ASTM C109 [21]. The average value was determined from six samples.

Before the XRD, TG, FTIR, and SEM analysis, the cured samples were immersed in absolute ethanol for 24 h to prevent further hydration, followed by placing them in a vacuum-drying oven at 50 °C for 24 h. During the XRD (Ultima IV) testing process, the accelerated voltage was set to 40 kV with a step width of 5 °/min. The degree interval was set to 5–80°. The TG (TGA55) test was carried out with an airflow rate of 20 mL/min and a temperature range from 30 to 800 °C in an N_2_ atmosphere. Moreover, the silicate groups of geopolymer were characterized by the FTIR (Bruker VERTEX 70, Bruker, Billerica, MA, USA). The infrared spectrum was collected with a frequency range of 4000–400 cm^−1^ with a resolution of 0.4 cm^−1^. In addition, the sample was coated with gold before being placed on the SEM (Zeiss Gemini300, ZEISS, Oberkochen, Germany) to observe the morphology and composition of the geopolymer, and the acceleration voltage was set as 15.0 kV. The characterization of experimental raw materials was also carried out using the relevant instruments mentioned above, except that the LS13320 laser-particle-size analyzer (Beckman Coulter, Shanghai, China) was used for the particle-size testing of the raw materials.

## 3. Results and Discussion

**Figure 4:**
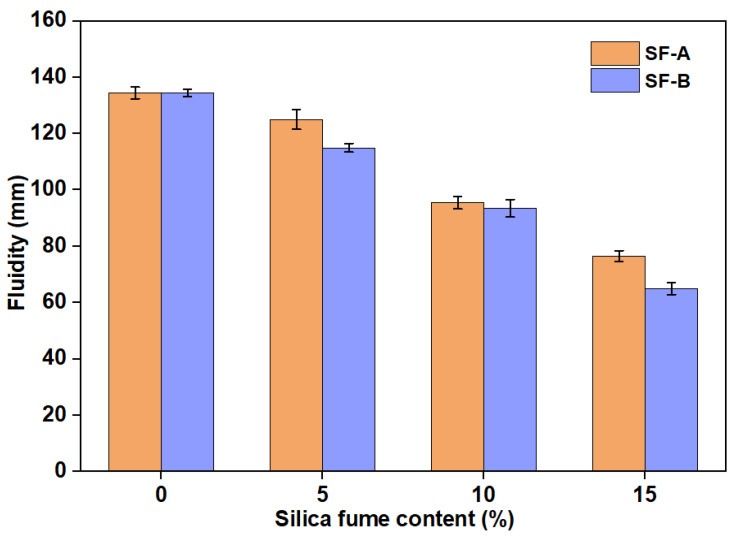
Workability of geopolymer samples modified with different SFs.

Although a few studies claimed that the addition of SF might be beneficial to the workability of fly ash-based geopolymer due to the spherical shape of SF [12,13,14], this study reveals an opposite phenomenon without changing the shape of the SFs. The workability results of the fly ash replacement by the SFs are shown in Figure 4. It can be observed that the replacement of fly ash by SFs leads to a constant workability reduction in the geopolymers due to the small particle size and large specific surface area of the SFs. With 15% replacement, the workability of SF-A replacement is 77 mm, while the SF-B is only 65 mm. Though both types of SFs can react rapidly in alkaline solutions to generate gel structure, the workability reduction rate of SF-B is higher than that of SF-A, indicating a potentially faster reaction rate through alkali activation.

**Figure 5:**
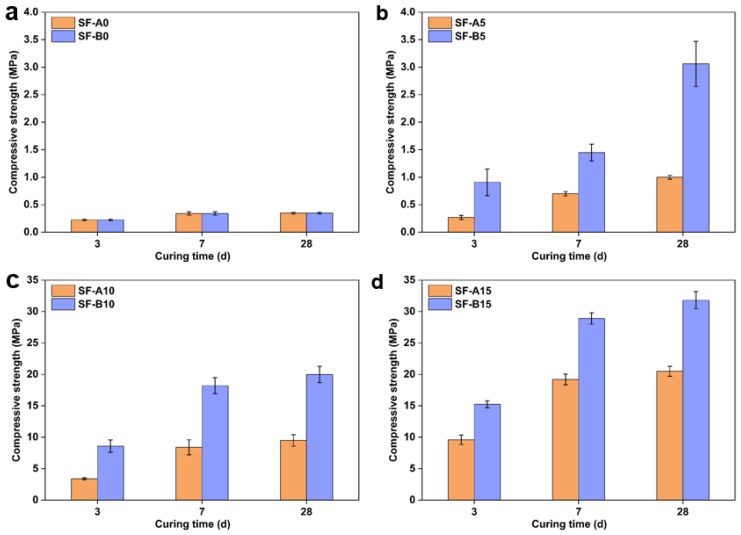
Mechanical properties of geopolymers with different SF contents: (**a**) 0%; (**b**) 5%; (**c**) 10%; (**d**) 15%.

Apart from the workability, the compressive strength of fly ash-based geopolymers modified with two types of SFs is shown in Figure 5. It can be observed that the addition of SFs is beneficial to enhancement of mechanical properties. Without the addition of SFs, the compressive strength of the fly ash-based geopolymer was negligible (below 0.5 MPa), while with the addition of a small amount of SFs, the compressive strength of the samples shows an observable enhancement. When the SF content increased to 5%, the compressive strength of SF-A5 reached 1 MPa, which increased by 1.9 times compared to SF-A0 (0.34 MPa). When the dosage of SF rose to 10% (SF-A10), the compressive strength increased about nine times compared to SF-A5 and reached 9.5 MPa. Upon further increasing the SF content to 15% (SF-A15), the compressive strength of the samples increased to 20.5 MPa (an additional 1.2 times higher compared to SF-A10).

Compared with SF-A, the reinforcement effect of SF-B is more distinctive. With 5% replacement of fly ash by the SF-B, the compressive strengths of SF-B5 increased by eight times (3.06 MPa) compared to SF-B0 (0.34 MPa), and the SF-B10 and SF-B15 reached 19.98 MPa and 31.81 MPa, respectively. The rapid enhancement of compressive strength suggests the rapid dissolution of SFs in alkaline environments proves its high activity, which helps to form silicate ions, participate in reactions, make condensation reactions more complete, and generate more hydration products. During the polymerization reaction, silicon and aluminum oxygen tetrahedra share oxygen atoms, constructing a complex three-dimensional network structure [22]. In addition, sodium ions in water glass are mainly distributed in the holes in gel network, which helps to form N-A-S-H gel [23], thus further improving the compressive strength. Under standard curing conditions, the compressive strength of the SF-B modified sample can reach as high as 31 MPa after being cured for 28 days. This growth trend is attributed to the continuous dissolution of SFs in alkaline pore solutions, forming additional polycondensation products. The combination of SF and fly ash has a good synergistic effect. From a physical perspective, unreacted SF and fly ash particles can be used to fill nanometer- and micrometer-sized voids, optimize raw material grading, improve pore structure, and reduce connected pores in geopolymers [24]. From the chemical point of view, the SF exerts pozzolanic activity in the early stage, producing more N-A-S-H gel [25], making up for the lack of low early activity of fly ash; and fly ash exhibits volcanic ash activity in the later stage, making the combination of SF and fly ash more complete in the entire hydration process. Although the results reveal no surprising modification effect, the visible higher modification effect of SF-B than SF-A needs to be explained because they look like they have similar size, morphology, and chemical compositions.

**Figure 6:**
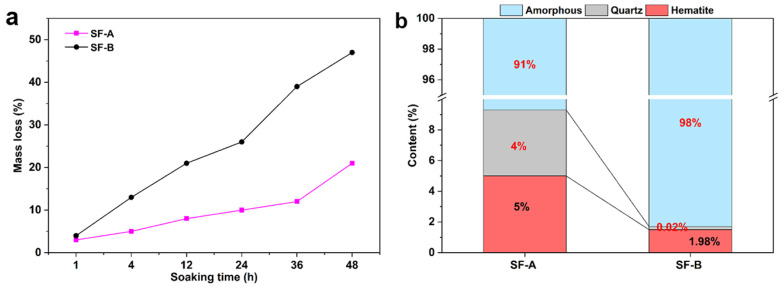
Mass loss (**a**) and QXRD results (**b**) of SF-A and SF-B after soaking in alkaline solution.

To unveil the difference between SF-A and SF-B, these two types of SFs were soaked in NaOH solution to investigate their activity in an alkali environment. Figure 6a indicates the mass loss of these two types of SFs in a NaOH solution as a function of soaking time. It can be observed that the mass loss of both SFs increases linearly with the elongation of soaking time. After 48 h of soaking, the mass loss of SF-A reached 21% while the mass loss of SF-B was 47%, indicating that more content of the SF-B had been dissolved. The results of the quantitative XRD analysis (Figure 6b) demonstrate that the amorphous content in SF-B samples was as high as 98%, while the SF-A was 91% with about 4% of the quartz remaining. Combined with the discovery of mass loss, it further confirms that SF-B samples have higher reactivity in alkaline solutions.

**Figure 7:**
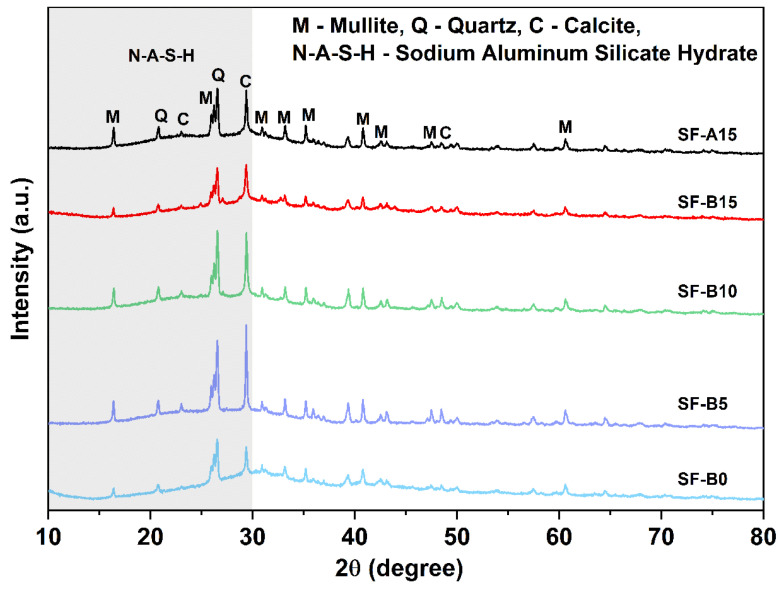
XRD patterns of geopolymer binders with various SF contents.

When solid silicate precursors (such as fly ash, limestone, metakaolin, etc.) react with alkaline activators (such as sodium hydroxide), the primary products of the hydration reaction are N-A-S-H gel and C-A-S-H gel, which are crucial components contributing to the mechanical strength of the system. It is noteworthy that the formation of C-A-S-H gel requires a high calcium content precursor as the calcium resource [22]. Considering the precursors employed in this test, limestone is the only calcium resource that can be used to form the C-S-H or C-A-S-H gels. However, given the limited solubility of calcite in an alkali environment, it might be reasonably inferred that the predominant gel type should be N-A-S-H gel. Figure 7 illustrates the XRD patterns of the sample after being cured for 28 d. In the XRD patterns, the N-A-S-H gel can be detected as a dispersion peak within the range of 10° to 30° (2θ). Furthermore, the presence of calcite peaks can be observed in all samples. However, a broad hump observed at 30° (2θ) representing the C-A-S-H cementitious phase [26] can be observed as well, and as the increase in SF content, the diffraction peak intensity of calcite (at 29°) and mullite (at 42°and 61°) exhibited a gradual reduction, indicating the increasing content of amorphous calcium phases. This phenomenon conflicts with the understanding that calcite is not soluble in an alkali environment.

The formation of N-A-S-H, C-S-H, and C-A-S-H gels in an alkali environment is a complex process involving multiple chemical reactions between substances [27]. Once activated by alkali, the polymerization of silicate and aluminate ions with sodium ions results in the formation of N-A-S-H gel. However, to form amorphous calcium-based gels, the activation of calcite is more complicated than the silica. Normally, the solubility of calcite in an alkali environment is quite limited, and thus the formation of the amorphous calcium-based gels will be thwarted due to the lack of soluble calcium ions. Different from the solution reaction mechanism, which is an isolated system, the calcite reaction with alkali in geopolymer manufacturing is exposed to air with a large amount of CO_2_. As a result, the soluble calcium ions can be provided based on the following equation:

CaCO_3_ + CO_2_ + H_2_O = Ca^2+^ + 2HCO_3_^−^ (1)

In this reaction mechanism, the pH value exerts a significant influence [28]. As the concentration of active silica increases, the intensity of the calcite diffraction peaks considerably declines due to the rising of the Si/Ca ratio, leading to the formation of C-A-S-H and N-A-S-H phases and the enhancement of mechanical properties.

**Figure 8:**
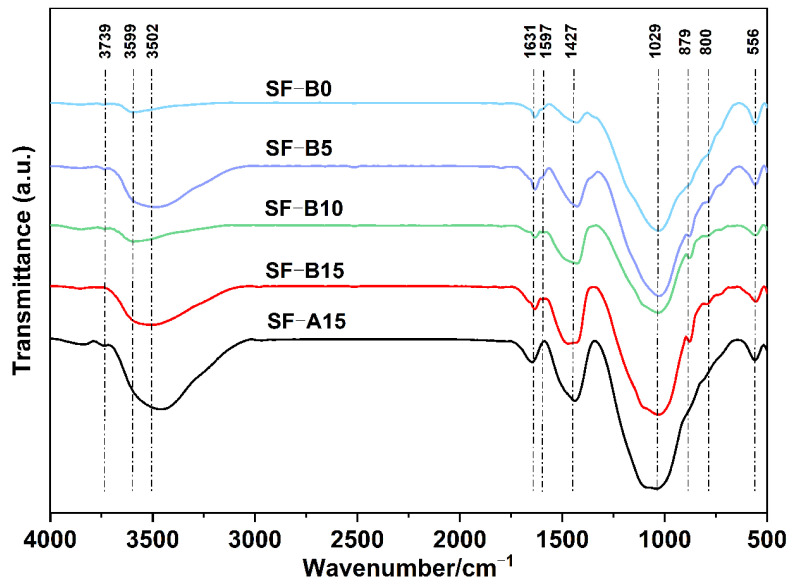
FTIR of different SF-doped amounts.

To further prove the structure of the amorphous gel phases, the FTIR results can be used as evidence to demonstrate the chemical bondings between Si, Ca, Al, and O. As demonstrated in Figure 8, the wide asymmetric absorption bands in the 1300–850 cm^−1^ and 500–420 cm^−1^ typically attribute to the tensile and flexural vibrations of Si–O–Si and Si–O–Al. The absorption peaks situated at approximately 556 cm^−1^, 800 cm^−1^, and 1029 cm^−1^ are associated with the vibration of the Si–O bond. The appearance of these peaks indicates the presence of N-A-S-H gels, as previously observed in previous studies [29,30,31]. The shift of the spectral band near 1029 cm^−1^ towards lower wavenumbers is attributed to the asymmetric tensile vibration of Si–O–Si and Al–O–Si in the semicrystalline cementitious phase of the product [32]. This spectral band is considered a pivotal indicator, providing insights into the extent of reaction in an alkali-activated fly ash-limestone powder system and the polymerization of hydration products [33]. The observed low wavenumber shift of the spectral band indicates that the introduction of SF-B enhances the reactivity of the alkali-activated fly ash-limestone powder system, and suggests that the degree of polymerization of the hydrated products increases. Meanwhile, the absorption peaks observed at 3739 cm^−1^, 3599 cm^−1^, and 3502 cm^−1^ are attributed to the tensile vibration of the O–H bond. This vibration is primarily associated with the vibration of hydroxyl groups present in the alkali activator and the hydration products. The absorption peak near 1631 cm^−1^ can be attributed to the vibration of the H–O–H bond of free water molecules in the system. The absorption peaks at 879 cm^−1^ and 1427 cm^−1^ are indicative of the vibrations of O–C–O bonds in carbonate ions, which are produced by the reaction of CO_2_ in the environment as indicated in Equation (1). The presence of these absorption peaks is corroborated by the XRD results, which serve to further substantiate the formation of the cementitious phases [34]. Based on our understanding of the cementitious network structure, we can further elucidate relevant information through thermogravimetric analysis.

**Figure 9:**
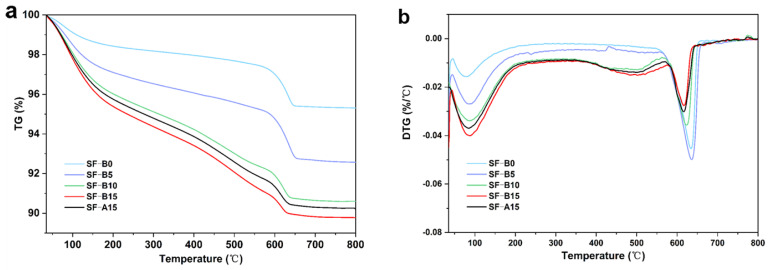
TG-DTG images of different SF dopants. (**a**) The TG images of samples with various proportions of SF substitution; (**b**) The DTG images of samples with various proportions of SF substitution.

**Figure 10:**
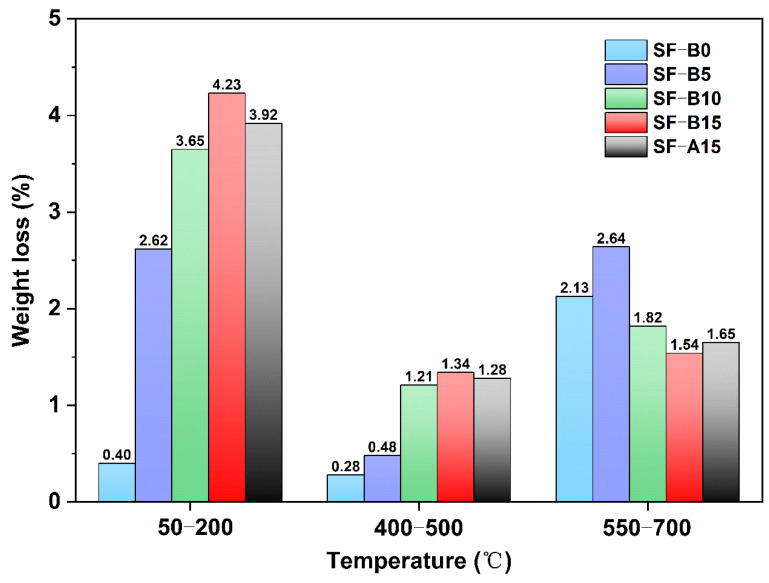
Weight loss curve of different SF dopants.

Figure 9a depicts the TG images of samples with various proportions of SF substitution. As the temperature rises from 30 °C to 800 °C, the sample undergoes decomposition, accompanied by the loss of water associated with the hydration products. The TG-DTG curve (Figure 9b) exhibits three distinct peaks. The initial peak occurs at approximately 100 °C, consistent with mass loss (as shown in Figure 10). This loss is primarily attributed to the evaporation of free water within the pores and the decomposition of bound water in the hydration products [35]. As illustrated in Figure 10, within the temperature range of 50 to 200 °C, the weight loss varies with fly ash replaced by SF. When the replacement reaches 15%, the maximum mass loss is 4.22%. The results demonstrated that the content of N-A-S-H gel increases significantly with the incorporation of SF-B, which well agrees with the strength-increasing phenomenon.

**Figure 11:**
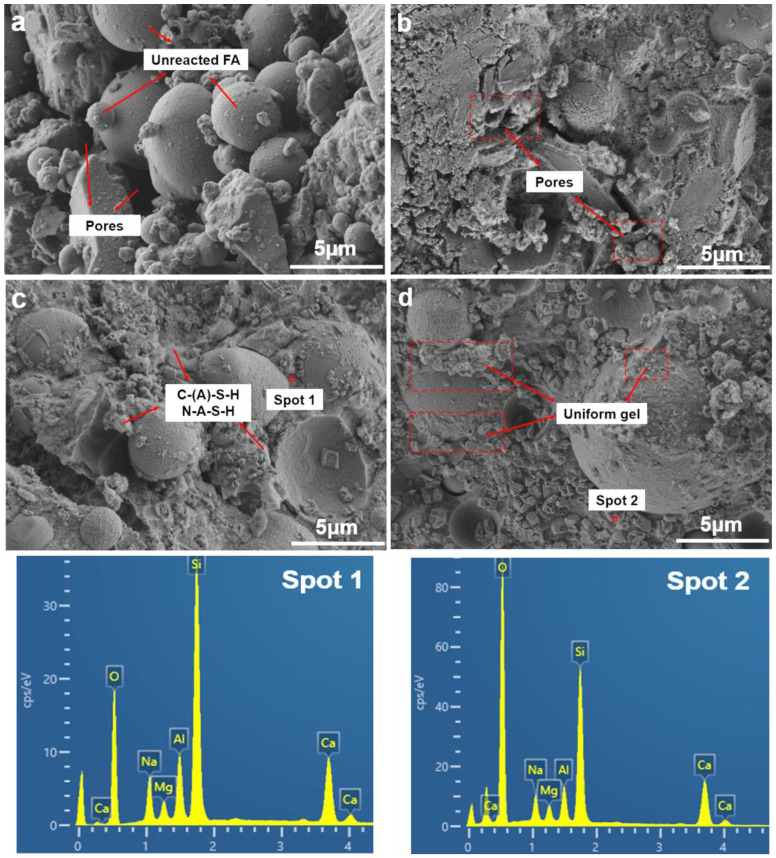
SEM images of different substitution ratios of SF. (**a**) SF-B0; (**b**) SF-B5; (**c**) SF-B10; (**d**) SF-B15.

To understand the microstructure evolution process, SEM morphologies of the 28-day samples are depicted in Figure 11. In Figure 11a, without any SF replacement, a large number of unreacted or partially reacted fly ash particles can be observed along with numerous pores and a relatively loose microstructure. This confirms the insufficient hydration reaction, which is consistent with the results of TG-DTG and explains the lowest compressive strength. With increasing contents of SF-B, the amount of unreacted fly ash particles significantly decreases, and dense hydration products can be observed. The continuous distribution of these hydration products significantly improves the density of the geopolymer. This phenomenon suggests that an appropriate amount of SF can effectively promote the hydration reaction, accelerate the formation of cementitious phases, and thus rapidly improve the compressive strength of the geopolymer. In Figure 11b–d, with a continuous increase in SF-B, a significant reduction in pores, a decrease in the number of unreacted particles, and a denser matrix structure can be observed. Although the silica content of SF-B is relatively lower than in previous studies (85% compared to 95%) [14,17], the high pozzolanic activity of SF-B provides a large amount of active silica content to the system, which serves as additional nucleation sites for hydration reactions and promotes the generation of hydration products. As proposed in ref. [36] and the aluminium-catalyzed oligomerization mechanism, Al substitution for Si in the gel network enhances cross-linking density, thereby improving mechanical properties. This aligns with our observation of denser N-A-S-H gels in SF-B-modified samples.

**Figure 12:**
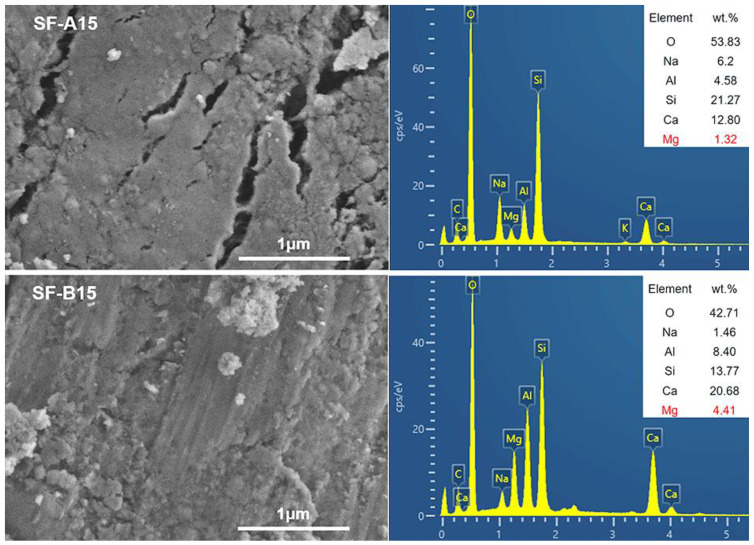
EDS area scan energy spectrum of hydration products.

The high-magnification SEM/EDS results are shown in Figure 12. As demonstrated in this figure, multiple cracks appeared on the surface of the SF-A15 sample due to the incompleteness of the hydration reaction. On the contrary, the surface of the SF-B15 sample exhibits a denser and crack-free structure, which is consistent with the SEM test results in Figure 11, indicating that SF-B15 has higher density and fewer pores. By comparing the two samples through EDS analysis, it was found that the Mg content in SF-B15 was significantly higher than that in SF-A15. This result suggests that the Mg element may play an important role in the formation of hydration products, replacing some metal cations and occupying corresponding spatial positions in the three-dimensional network structure. This substitution effect may help generate more stable and dense hydration products, thereby enhancing the overall structural strength of the sample. Therefore, the EDS analysis results further elucidate the micro mechanism of SF-B15 samples having higher compressive strength. The appearance of Mg not only promotes the hydration reaction but also optimizes the three-dimensional network structure of the sample by changing the chemical composition of the hydration products, resulting in higher compressive strength and better overall performance at the macroscopic level.

The unique properties of SF-A and SF-B in geopolymer systems are mainly determined by their crystallinity, chemical composition, and reactivity in alkaline environments. As shown in Figure 6b, the amorphous content of SF-B is 98%, significantly higher than SF-A at 90%, and the higher amorphous silicon dioxide content in SF-B can dissolve faster in alkaline activators. By observing Figure 6a, it is found that the weight loss of SF-B is 47% and that of SF-a is 21% after soaking in NaOH for 48 h. The rapid dissolution of SF-B releases more silicate ions, accelerates the formation of N-A-S-H gel, and enhances the cross-linking in the geopolymer matrix. In combination with the EDS analysis in Figure 12, it is found that SF-B contains 4.41 wt.% Mg, while SF-A contains very little Mg, indicating that magnesium ions may replace aluminum or calcium in the gel network to stabilize the N-A-S-H structure, thus promoting the formation of more dense hydration products. By comparison, it can be observed that the crystalline quartz phase of SF-A can act as an inert filler, limiting the progress of alkali activation reactions, resulting in a less dense microstructure as shown in Figure 11a–c.

**Figure 13:**
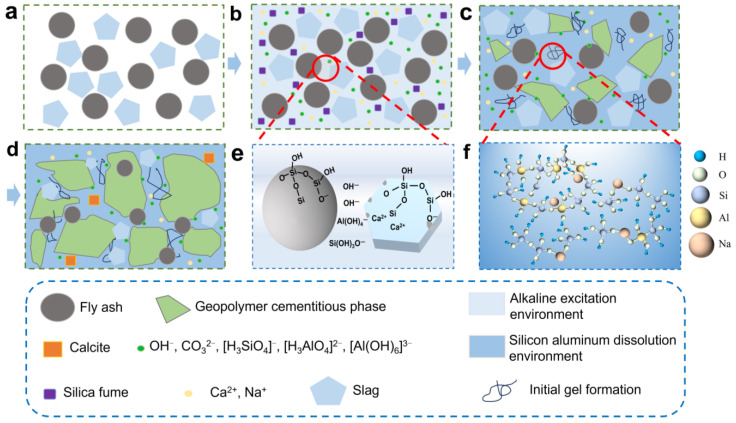
Schematic diagram of the polymerization mechanism of geopolymers: (**a**) initial stage, (**b**) dissolve, (**c**) polymerization, (**d**) solidify, (**e**) schematic diagram illustrating the dissolution of fly ash and limestone powder in an alkaline environment, (**f**) molecular structure diagram of the gel.

In the context of fixed limestone powders, the SF-B with the content of Mg along with high active silica content facilitates the compressive strength enhancement of geopolymers. The formation mechanism of the geopolymer is schematically illustrated in Figure 13. The alkaline environment of the geopolymer is provided by water glass following modulus adjustment, which serves to promote the dissolution of fly ash and limestone powders. In the presence of strong alkaline conditions, the aluminosilicate structure undergoes dissolution, resulting in the production of OH^−^, CO_3_^2−^, [H_3_SiO_4_]^−^, [H_3_AlO_4_]^2−^, and [Al(OH)_6_]^3−^ oligomers, along with other oligomers [37]. These oligomers subsequently form N-A-S-H gels. As illustrated in Figure 13e, the dissolution of Si and Al in an alkaline environment results in the gradual erosion of the spherical surface of fly ash. This process leads to a transformation in the polyhedral network structure of Si–O and Al–O, whereby the network structure of Si–O–Si and Al–O–Si is destroyed, primarily forming [SiO_4_] and [AlO_4_]^−^. As a consequence of the dissolution of the aluminosilicate structure, Si and Al are released into the solution, where they form oligomers such as [H_3_SiO_4_]^−^, [H_3_AlO_4_]^2−^, and [Al(OH)_6_]^3−^, as illustrated in Equations (2)–(4). The oligomers combine with sodium ions provided by water glass to form nucleation sites, which can be readily re-polymerized at room temperature [27]. They diffuse from the raw material surface to the pores for condensation polymerization, forming crystals or cementitious material, as illustrated in Equation (5). The aluminosilicate structures are rearranged and reorganized to form a large three-dimensional network structure, as illustrated in Figure 13f.

(2) $${\mathrm{SiO}}_{2}+{\mathrm{OH}}^{-}+H_{2}O\;\to \;{\left[H_{3}{\mathrm{SiO}}_{4}\right]}^{-}$$

(3) $${\mathrm{AlO}}_{2}^{-}+{\;\mathrm{OH}}^{-}+H_{2}O\;\to \;{\left[H_{3}{\mathrm{AlO}}_{4}\right]}^{2-}\;$$

(4) $${\mathrm{AlO}}_{2}+{\mathrm{OH}}^{-}+H_{2}O\;\to \;{\left[\mathrm{Al}{\left(\mathrm{OH}\right)}_{6}\right]}^{3-}$$

(5) $${\mathrm{Na}}^{+}+{\left[H_{3}{\mathrm{SiO}}_{4}\right]}^{-}+{\left[H_{3}{\mathrm{AlO}}_{4}\right]}^{2-}\;\to \;N\text{-}A\text{-}S\text{-}H$$

With the extension of the curing time, N-A-S-H gels were observed to adhere to the surface of the raw material, with the interconnections between the gel networks becoming more pronounced. The continuous occurrence of geopolymerization reactions leads to the interconnection of newly generated products, forming a highly dense three-dimensional aluminosilicate network structure of geological polymers. In the alkali-activated fly ash-limestone powder system, this three-dimensional network structure can significantly enhance the mechanical properties of geopolymers.

## 4. Conclusions

In this study, two types of silica fumes were used as a substitute to enhance the alkali-activated fly ash-limestone powder system. The effect of SF on the workability and compressive strength of an alkali-activated fly ash-limestone powder system was studied. The hydration products were comprehensively characterized using X-ray diffraction (XRD), Fourier transform infrared spectroscopy (FTIR), derivative thermogravimetric analysis (DTG), thermogravimetric analysis (TGA), and scanning electron microscopy energy dispersive X-ray spectroscopy (SEM-EDS) to reveal reaction mechanisms and the products that control the final strength development of geopolymers.

(1) The addition of SF can fill the pores and promote the formation of hydration products during the reaction process, improving compressive strength. Compared with a 60 wt.% fly ash and 40 wt.% limestone powder system, using 15 wt.% SF instead of fly ash significantly increased compressive strength by over 30 times.
(2) Due to the small particle size and large specific surface area of SF, the interaction between particles is increased, resulting in a gradual decrease in workability.
(3) The microstructure analysis results indicate that the SF is beneficial for activating fly ash and forming an N-A-S-H cementitious phase. Compared with SF with similar particle size and chemical composition, SF-B has a higher content of amorphous silica, and the generated cementitious material is more closely combined with partially reacted fly ash particles, showing a fairly strong strengthening effect.

The findings support the application of SF-modified geopolymers in high-strength construction components (e.g., precast blocks, structural columns) and waste-to-resource initiatives, promoting sustainable infrastructure development.

## Figures and Tables

**Table 1:** Chemical composition of raw materials (wt.%).

	SiO_2_	Al_2_O_3_	CaO	Fe_2_O_3_	MgO	MnO	Na_2_O	K_2_O	SO_3_	P_2_O_5_	Others
Fly ash	54.80	29.72	4.35	4.26	0.67	0.03	0.65	2.13	0.72	0.33	2.34
Limestone powders	11.36	15.66	56.88	2.75	1.32	0.03	0.09	0.72	9.45	0.08	1.66
SF-A	80.07	0.67	4.13	6.61	1.36	0.05	1.31	0.61	0.36	0.13	4.7
SF-B	84.77	0.49	0.81	1.48	8.26	0.11	2.37	0.94	0.23	0.08	0.46

**Table 2:** Mixing proportion of geopolymer.

Sample Number	FA (%)	LS (%)	SF-A (%)	SF-B (%)	H_2_O (%)	NaOH (%)	WG (%)
SF-A0	60	40	0	0	32.1	2.5	12.6
SF-A5	55	40	5	0	32.1	2.5	12.6
SF-A10	50	40	10	0	32.1	2.5	12.6
SF-A15	45	40	15	0	32.1	2.5	12.6
SF-B0	60	40	0	0	32.1	2.5	12.6
SF-B5	55	40	0	5	32.1	2.5	12.6
SF-B10	50	40	0	10	32.1	2.5	12.6
SF-B15	45	40	0	15	32.1	2.5	12.6

## Data Availability

The original contributions presented in this study are included in the article. Further inquiries can be directed to the corresponding authors.
